# Oral microbiome diversity shapes the association between leisure-time physical activity and cognitive function among older adults

**DOI:** 10.1016/j.isci.2026.115086

**Published:** 2026-02-19

**Authors:** Yanwei You, Yicong Cui, Kefeng Zheng, Haopeng Yang, Chuanwen Yu, Qingyuan Wang, Yuquan Chen, Xindong Ma

**Affiliations:** 1 Division of Sports Science & Physical Education, Tsinghua University, Beijing 100084, China; 2 Saw Swee Hock School of Public Health, National University of Singapore, Singapore 117549, Singapore; 3 Beijing Hospital, National Center for Gerontology, Beijing 100730, China; 4 College of Physical Education and Health, Heze University, Heze 274015, China; 5 Chinese Academy of Medical Sciences and Peking Union Medical College, Beijing 100730, China; 6 IDG/McGovern Institute for Brain Research, Tsinghua University, Beijing 100084, China

**Keywords:** Health informatics, health sciences, medical specialty, medicine, psychiatry

## Abstract

Regular leisure-time physical activity and the composition of the oral microbiome may influence late-life cognition, but their combined effects are unclear. We studied 575 US adults aged 60–69 years from a nationally representative survey with oral microbiome sequencing, self-reported leisure-time physical activity, and four standard cognitive tests of memory, verbal fluency, and processing speed. Higher weekly physical activity and meeting current guideline levels were associated with better memory and processing speed scores after multivariable adjustment. Greater oral microbiome diversity, including higher richness and evenness, was also related to better processing speed. Among participants who met activity guidelines, those with intermediate to high Shannon diversity and a specific community composition profile showed the most favorable verbal fluency and processing speed. These findings suggest that maintaining both regular physical activity and a diverse oral microbiome may jointly support cognitive function in later life and promote healthy aging.

## Highlights

• Nationally representative study linking LTPA, oral microbiome, and cognition
• Greater oral α-diversity is independently associated with better cognitive function
• Physical activity-cognition link is stronger in diverse, commensal-rich communities
• Findings support integrated lifestyle and oral health strategies for healthy aging

## Graphical abstract

**Figure:**
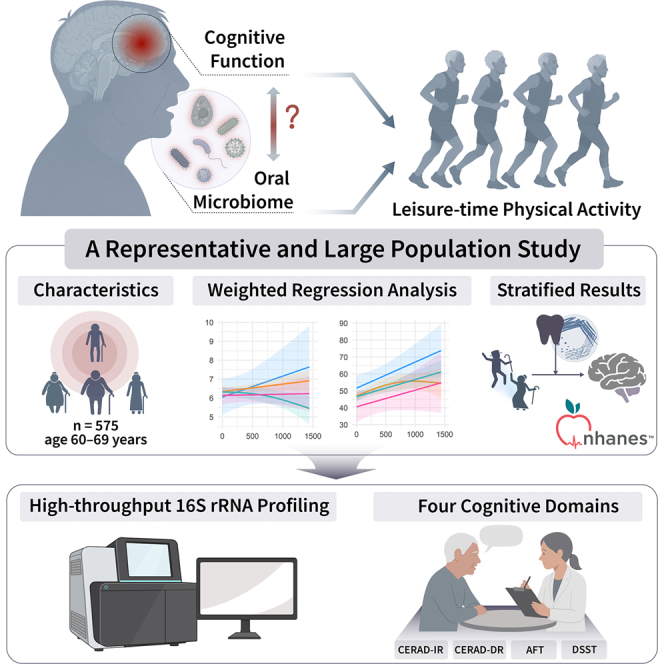

## Introduction

The accelerating rise in cognitive impairment and dementia constitutes a major public-health emergency: The Global Burden of Disease 2019 forecast projects the number of affected individuals to swell from ∼57 million in 2019 to more than 150 million by 2050, imposing an ever-growing societal and economic toll.^1^ In the absence of effective curative treatments, early identification and prevention-based strategies have become critical approaches for mitigating the rising prevalence of cognitive disorders.^2^ Although chronological aging is the dominant non-modifiable risk factor,^3^ mounting evidence indicates that lifestyle and biological factors offer leverage points for delaying or attenuating phenotypic aging and cognitive decline.^4^^,^^5^^,^^6^

Leisure-time physical activity (LTPA) is among the modifiable lifestyle-related risk behaviors. Unlike long-term dietary restructuring or sleep improvement constrained by circadian rhythms, physical activity features low intervention costs and high sustainability, making it a prominent focus in cognitive health research and a clinically recognized adjunctive strategy to promote physical and mental well-being among older adults.^7^^,^^8^ A 2024 JAMA Network Open meta-analysis synthesizing 104 longitudinal cohorts (≈341 000 participants) demonstrated that every standard-deviation increase in baseline activity predicted a 3%–4% slower rate of cognitive decline over follow-up, albeit with considerable heterogeneity and small absolute effects.^9^ Randomized controlled trials corroborate a causal benefit: a 2023 meta-analysis of exercise interventions in adults ≥50 years reported a pooled standardized mean difference of ∼0.29 in global cognition compared with non-exercise controls, with aerobic, resistance and multicomponent programs all conferring gains.^10^ Trajectory modeling from the CHARLS cohort indicates a high degree of synchrony between changes in physical activity and cognitive function in adults over 55, supporting a dynamic and bidirectional association.^11^ Mechanistically, endurance exercise enhances cerebral perfusion, boosts brain-derived neurotrophic factor, elevates lactate signaling, and dampens chronic inflammation—pathways now recognized as core neuroprotective mechanisms.^12^^,^^13^ Yet effect sizes vary widely and null findings persist, implying the presence of unmeasured effect-modifying factors.

The oral microbiome has emerged as a novel biological modifier. It can exert direct influence on the nervous, immune, and circulatory systems, or indirectly disrupt gut microbiota and immune homeostasis, thereby impacting the brain and cognitive processes.^14^ A 2025 systematic review of 21 studies identified a consistent dysbiotic signature in Alzheimer’s disease (enrichment of Porphyromonas, Fusobacterium, Tannerella and other periodontitis-related taxa, alongside the depletion of commensal genera such as Streptococcus and Gemella) and noted that cognitively impaired adults often exhibit lower oral bacterial α-diversity.^15^ The oral cavity harbors a remarkably complex community—now estimated to exceed 700 bacterial, archaeal and fungal taxa—and its ecological status is typically summarized along two complementary axes: α-diversity, which captures the richness and evenness within a single sample, and β-diversity, which describes compositional differences between samples.^16^ Previous work shows that reduced α-diversity accompanies periodontitis, while abnormally high values have been observed in frail older adults, suggesting that both extremes may reflect ecological dysregulation with clinical relevance.^17^^,^^18^ Large population studies further report that inter-individual variation in β-diversity tracks oral pre-cancer, predicts incident cardiovascular events, and correlates with faster cognitive decline, underscoring the potential of community-level signatures as early markers of systemic disease risk.^19^^,^^20^^,^^21^ In addition, recent analyses of older adults have linked variation in oral α- and β-diversity, as well as periodontitis-related taxa, to lower composite cognitive scores and less favorable profiles of Alzheimer’s-related biomarkers in community-based samples and MCI cohorts.^22^ Taken together, current evidence suggests that the oral microbiome may contribute to neurodegenerative disease primarily through oral-brain pathways and, to a lesser extent, possibly through oral-gut-brain routes via its impact on the gut microbiota^23^^,^^24^; however, these gut-related pathways are less well established and should be regarded as tentative.

Physical activity and the oral microbiome are not independent. Recent evidence suggests that physical activity may influence cognitive function not only through metabolic and neurophysiological pathways, but also by modulating the oral microbiome. A 16-week randomized trial in older adults confirmed that structured exercise can remodel oral ecosystems. Specifically, moderate-intensity continuous training increased salivary Shannon diversity and shifted the Firmicutes-to-Bacteroidetes ratio, whereas high-intensity interval training produced complementary but distinct compositional changes.^25^ Observational analyses further suggest that physically active individuals have a ∼23% lower risk of periodontitis, a canonical manifestation of oral dysbiosis,^26^ supporting the possibility that LTPA may confer protective effects partly via improving oral microbial balance. Collectively, these data raise the possibility that a favorable oral microbiome could amplify—while dysbiosis might blunt—the cognitive dividends of physical activity. However, no nationally representative study has yet tested whether oral microbial diversity moderates LTPA-cognition associations, leaving a gap in our understanding of lifestyle-microbiome synergies for brain aging.

To address this gap, we leveraged data from the 2011–2012 National Health and Nutrition Examination Survey (NHANES), which uniquely integrates high-throughput 16S rRNA profiling of oral rinse samples with detailed LTPA questionnaires, standardized cognitive testing, and extensive covariate information in a representative U.S. older-adult sample. Our aims had three aspects: (i) to quantify associations between LTPA and four cognitive domains, (ii) to evaluate independent links between oral microbiome diversity and cognition, (iii) to assess whether oral α- and β-diversity moderate the LTPA-cognition relationship, and (iv) to explore an inflammation-related mechanism using white blood cell (WBC) count as an available biomarker. We hypothesized that greater microbial diversity would enhance—whereas lower diversity would attenuate—the cognitive advantages of higher physical activity.

## Results

### Characteristics of study participants

A total of 575 participants aged 60 to 69 years from the NHANES 2011–2012 cycle were included in the final analysis. All participants had complete data on oral microbiome and cognitive assessments. Table 1 summarizes their weighted baseline characteristics. The average age of the sample was 63.8 ± 2.8 years, and approximately half of the participants were male. In terms of race and ethnicity, 30.6% identified as non-Hispanic White, 34.6% as non-Hispanic Black, 9.9% as Mexican American, and 24.9% as other racial or ethnic backgrounds. Over half of the participants (54.4%) had attained a college-level education or higher, while 11.3% had not completed high school. Regarding socioeconomic status, 19.1% of the sample had a poverty-income ratio (PIR) below 1, while 41.6% had a PIR ≥3. About half of the participants (51.3%) reported never smoking, and 47.3% reported moderate alcohol consumption. Regarding LTPA, 320 participants (55.7%) reported no qualifying LTPA (0 MET-min·week^−1^), 86 (15.0%) accumulated 1–<600 MET-min·week^−1^, and 169 (29.4%) achieved ≥600 MET-min·week^−1^. Obesity was common, with 42.4% of participants having a BMI ≥30. The prevalence of diabetes and hypertension was 34.3% and 35.0%, respectively, and 15.1% of participants had a history of cardiovascular disease. The mean scores for cognitive tests were as follows: 6.4 ± 1.9 for CERAD immediate recall, 6.2 ± 2.1 for CERAD delayed recall, 17.4 ± 5.6 for the Animal Fluency Test (AFT), and 50.0 ± 17.7 for the Digit Symbol Substitution Test (DSST).

**Table 1:** Characteristics of eligible participants in this study

Variables	All participants
Age (years)	63.8 ± 2.8
Male sex (%)	49.7
Race/ethnicity (%)	
Non-hispanic White	30.6
Non-hispanic Black	34.6
Mexican American	9.9
Other Race/ethnicity	24.9
Educational level (%)	
Below high school	11.3
High school	34.3
College or above	54.4
Marital status	
Never married	8.1
Married/living with partner	58.4
Widowed/divorced	33.5
Poverty income ratio	
< 1	19.1
[1,3)	39.3
≥ 3	41.6
Smoke status (%)	
Never smoking	51.3
Former smoking	31.7
Current smoking	17.0
Alcohol use status (%)	
None	41.7
Moderate alcohol use	47.3
High alcohol use	11.0
Meeting LTPA guidelines (%)	
No	45.6
Insufficient (1–600 MET)	25.0
Sufficient (>600 MET)	29.4
BMI (%)	
< 25	23.5
[25, 30)	34.1
≥ 30	42.4
Diabetes (%)	
No	65.7
Yes	34.3
Hypertension (%)	
No	65.0
Yes	35.0
CVD (%)	
No	84.9
Yes	15.1
Cognitive function	
CERAD-IR	6.4 ± 1.9
CERAD-DR	6.2 ± 2.1
AFT	17.4 ± 5.6
DSST	50.0 ± 17.7

Notes: Weighted percentage for category variables and weighted mean ± standard deviations for continuous variables: BMI, body mass index; LTPA, leisure-time physical activity; CERAD-DR, Consortium to Establish a Registry for Alzheimer’s Disease-delayed recall; CERAD-IR, Consortium to Establish a Registry for Alzheimer’s Disease-immediate recall; AFT, Animal Fluency Test; DSST, DigitSymbol Substitution Test.

### Leisure-time physical activity and cognitive function

In the fully adjusted models—summarized separately for each cognitive domain—greater LTPA was linked to superior performance across the board (Tables 2, 3, S1, and S2). Each 100-MET-min·week^−1^ increment in LTPA corresponded to higher scores on CERAD immediate recall (β = 0.013, 95% CI 0.002–0.023, *p* = 0.019), CERAD delayed recall (β = 0.023, 95% CI 0.010–0.036, *p* = 0.002), animal fluency (AFT; β = 0.056, 95% CI 0.003–0.109, *p* = 0.040); for DSST, the coefficient suggested a positive but non-significant trend toward higher scores (β = 0.171, 95% CI -0.010–0.351, *p* = 0.062). Table 2 details these associations for AFT, incorporating LTPA, four alpha-diversity indices, and three beta-diversity measures within weighted linear models; Table 3 presents the parallel results for DSST. Table S1 provides the corresponding estimates for CERAD immediate recall (CERAD-IR), while Table S2 reports those for CERAD delayed recall (CERAD-DR).

**Table 2:** Association results of leisure time physical activity, four α-diversity measures, three beta-diversity measures, and AFT using weighted linear regression models

		Crude model		Adjusted Model	
		β (95% CI)	*p-value*	β (95% CI)	*p-value*
Leisure-time physical activity	Continuous (Per 100-MET increase)	0.055(−0.002, 0.112)	0.057	0.056(0.003, 0.109)	0.040
	No	Reference		Reference	
	Insufficient (1–600 MET)	2.564(0.700, 4.428)	0.011	2.572(0.559, 4.584)	0.016
	Sufficient (>600 MET)	2.149(0.319, 3.979)	0.024	2.144(0.352, 3.936)	0.022
OTUs	Continuous	−0.004(−0.031, 0.023)	0.752	−0.005(−0.031, 0.021)	0.704
	Q1 (<80.20)	Reference		Reference	
	Q2 (80.20–101.08)	0.29(−1.773, 2.353)	0.765	0.267(−1.744, 2.279)	0.780
	Q3 (101.08–126.71)	−0.11(−1.886, 1.665)	0.895	−0.133(−1.838, 1.573)	0.870
	Q4 (>126.71)	−0.081(−3.758, 3.597)	0.963	−0.16(−3.627, 3.306)	0.922
FaPhyloDiv	Continuous	−0.101(−0.387, 0.184)	0.459	−0.107(−0.379, 0.164)	0.414
	Q1 (<10.58)	Reference		Reference	
	Q2 (10.58–12.32)	−0.411(−1.917, 1.095)	0.563	−0.401(−1.911, 1.109)	0.578
	Q3 (12.32–14.34)	0.384(−2.646, 3.413)	0.787	0.349(−2.676, 3.373)	0.808
	Q4 (>14.34)	−1.138(−3.460, 1.183)	0.306	−1.203(−3.427, 1.021)	0.265
InvSimpson	Continuous	2.034(−12.567, 16.635)	0.770	1.804(−12.926, 16.535)	0.798
	Q1 (<0.88)	Reference		Reference	
	Q2 (0.88–0.91)	0.373(−0.944, 1.691)	0.549	0.407(−0.967, 1.780)	0.536
	Q3 (0.91–0.93)	−0.028(−2.291, 2.234)	0.979	−0.035(−2.292, 2.223)	0.974
	Q4 (>0.93)	0.299(−2.764, 3.363)	0.835	0.269(−2.737, 3.274)	0.851
ShanWienDiv	Continuous	0.213(−1.279, 1.705)	0.764	0.184(−1.260, 1.627)	0.791
	Q1 (<4.09)	Reference		Reference	
	Q2 (4.09–4.49)	−0.394(−2.094, 1.305)	0.622	−0.404(−2.065, 1.256)	0.610
	Q3 (4.49–4.93)	−0.929(−3.166, 1.308)	0.383	−0.972(−3.044, 1.101)	0.332
	Q4 (>4.93)	0.8(−1.757, 3.356)	0.509	0.709(−1.660, 3.078)	0.532
Braycurtis distance	Cluster 1	Reference		Reference	
	Cluster 2	2.544(−0.476, 5.565)	0.091	2.34(−0.712, 5.391)	0.122
	Cluster 3	−0.733(−2.361, 0.894)	0.346	−0.695(−2.307, 0.917)	0.371
	Cluster 4	−0.691(−7.379, 5.997)	0.826	−0.555(−7.396, 6.285)	0.864
Unweighed Unifrac	Cluster 1	Reference		Reference	
	Cluster 2	1.206(−0.321, 2.733)	0.111	1.387(−0.161, 2.935)	0.075
	Cluster 3	4.392(2.161, 6.622)	0.001	4.489(2.162, 6.815)	0.001
	Cluster 4	1.062(−1.474, 3.597)	0.380	1.206(−1.516, 3.928)	0.358
Weighed Unifrac	Cluster 1	Reference		Reference	
	Cluster 2	−3.838(−7.046, −0.630)	0.023	−4.409(−7.570, −1.249)	0.011
	Cluster 3	−4.149(−7.290, −1.009)	0.014	−4.414(−7.420, −1.408)	0.008
	Cluster 4	−5.784(−7.737, −3.831)	<0.001	−5.974(−7.872, −4.075)	<0.001

Notes: Crude model, no covariates were adjusted. Adjusted model, age, sex, race/ethnicity, body mass index, marital status, education, poverty income ratio, smoke status, alcohol use status, and chronic diseases were adjusted.

**Table 3:** Association results of leisure time physical activity, four alpha-diversity measures, three β-diversity measures, and DSST using weighted linear regression models

		Crude model		Adjusted Model	
		β (95% CI)	*p-value*	β (95% CI)	*p-value*
Leisure-time physical activity	Continuous (Per 100-MET increase)	0.155(−0.020, 0.329)	0.079	0.171(−0.010, 0.351)	0.062
	No	Reference		Reference	
	Insufficient (1–600 MET)	3.741(−2.243, 9.725)	0.201	3.981(−1.950, 9.913)	0.171
	Sufficient (>600 MET)	7.934(3.272, 12.596)	0.002	7.972(3.421, 12.524)	0.002
OTUs	Continuous	−0.002(−0.064, 0.060)	0.943	−0.017(−0.073, 0.038)	0.515
	Q1 (<80.20)	Reference		Reference	
	Q2 (80.20–101.08)	5.045(1.290, 8.799)	0.013	4.813(0.747, 8.878)	0.024
	Q3 (101.08–126.71)	1.044(−3.515, 5.604)	0.627	0.16(−4.100, 4.420)	0.937
	Q4 (>126.71)	1.102(−5.274, 7.479)	0.713	−0.35(−5.583, 4.884)	0.888
FaPhyloDiv	Continuous	−0.265(−1.010, 0.480)	0.458	−0.425(−1.119, 0.268)	0.212
	Q1 (<10.58)	Reference		Reference	
	Q2 (10.58–12.32)	2.063(−2.772, 6.898)	0.371	2.016(−2.675, 6.707)	0.372
	Q3 (12.32–14.34)	2.017(−3.197, 7.231)	0.416	0.346(−4.944, 5.635)	0.891
	Q4 (>14.34)	−1.566(−7.266, 4.135)	0.561	−2.803(−7.516, 1.909)	0.223
InvSimpson	Continuous	14.859(−24.672, 54.389)	0.434	8.393(−35.416, 52.201)	0.690
	Q1 (<0.88)	Reference		Reference	
	Q2 (0.88–0.91)	5.284(1.137, 9.431)	0.017	4.805(0.487, 9.123)	0.032
	Q3 (0.91–0.93)	2.682(−1.597, 6.960)	0.197	2.004(−3.016, 7.025)	0.406
	Q4 (>0.93)	3.842(−2.352,10.036)	0.201	3.391(−3.278, 10.061)	0.294
ShanWienDiv	Continuous	1.251(−1.760, 4.263)	0.388	0.765(−2.476, 4.005)	0.624
	Q1 (<4.09)	Reference		Reference	
	Q2 (4.09–4.49)	−0.16(−4.677, 4.356)	0.940	−0.646(−4.786, 3.493)	0.743
	Q3 (4.49–4.93)	1.445(−3.615, 6.504)	0.545	1.147(−4.005, 6.298)	0.640
	Q4 (>4.93)	1.262(−3.916, 6.439)	0.605	0.286(−5.042, 5.614)	0.910
Braycurtis distance	Cluster 1	Reference		Reference	
	Cluster 2	0.899(−9.709, 11.507)	0.858	4.091(−7.440, 15.622)	0.455
	Cluster 3	0.601(−2.925, 4.127)	0.702	0.606(−2.542, 3.755)	0.682
	Cluster 4	3.98(−9.663, 17.623)	0.540	1.369(−11.681, 14.418)	0.823
Unweighed Unifrac	Cluster 1	Reference		Reference	
	Cluster 2	7.507(−6.463, 21.478)	0.268	5.684(−8.011, 19.378)	0.384
	Cluster 3	13.216(4.653, 21.778)	0.005	10.585(2.454, 18.716)	0.015
	Cluster 4	6.535(−8.687, 21.757)	0.373	6.483(−9.043, 22.010)	0.381
Weighed Unifrac	Cluster 1	Reference		Reference	
	Cluster 2	−5.155(−10.860, 0.551)	0.073	−5.367(−12.148, 1.414)	0.111
	Cluster 3	−5.576(−11.948, 0.796)	0.082	−5.83(−13.118, 1.458)	0.108
	Cluster 4	−8.708(−16.980,-0.436)	0.040	−7.251(−15.235, 0.733)	0.072

Notes: Crude model, no covariates were adjusted. Adjusted model, age, sex, race/ethnicity, body mass index, marital status, education, poverty income ratio, smoke status, alcohol use status, and chronic diseases were adjusted.

When LTPA was analyzed categorically, participants who met or exceeded the guideline threshold of ≥600 MET-min·week^−1^ demonstrated markedly better cognition than physically inactive peers. Specifically, the “sufficient” group scored higher on DSST (β = 7.972, 95% CI 3.421–12.524, *p* = 0.002), AFT (β = 2.144, 95% CI 0.352–3.936, *p* = 0.022), and CERAD-DR (β = 0.548, 95% CI 0.033–1.062, *p* = 0.039), reinforcing the linear trend observed with the continuous exposure.

### Oral microbiome diversity and cognitive function

Among the four α-diversity measures, OTUs and InvSimpson were significantly associated with DSST scores (Table 3). In the adjusted models, participants in the second quartile of OTU richness (80.20–101.08) scored higher on the DSST compared to those in the lowest quartile (β = 4.813, 95% CI: 0.747 to 8.878, *p* = 0.024). Likewise, individuals in the second quartile of the InvSimpson index (0.88–0.91) showed better DSST performance (β = 4.805, 95% CI: 0.487 to 9.123, *p* = 0.032). No significant associations were observed between α-diversity measures and the other three cognitive domains.

As for β-diversity measures, in the adjusted models, significant associations were observed between β-diversity and cognitive function, particularly for the Animal Fluency Test (AFT) and DSST (Tables 2 and 3). For AFT, participants in Cluster 3, based on unweighted UniFrac distance, scored significantly higher than those in Cluster 1 (reference group) (β = 4.489, 95% CI: 2.162 to 6.815, *p* = 0.001). In contrast, across the same clustering, Cluster 4 derived from weighted UniFrac was associated with lower AFT scores (β = −5.974, 95% CI: −7.872 to −4.075, *p* < 0.001). Regarding DSST performance, individuals in Cluster 3 of the unweighted UniFrac classification also showed higher scores (β = 10.585, 95% CI: 2.454 to 18.716, *p* = 0.015). Additionally, participants in Cluster 4 of the weighted UniFrac profile demonstrated lower DSST scores compared to the reference group (β = −7.251, 95% CI: −15.235 to 0.733, *p* = 0.072), though this association did not reach statistical significance. However, no significant associations between β-diversity clusters and performance on the CERAD immediate or delayed recall tasks were observed across the three distance measures (Tables S1 and S2).

### Joint associations between α-diversity measures, physical activity, and cognitive function

**Figure 1:**
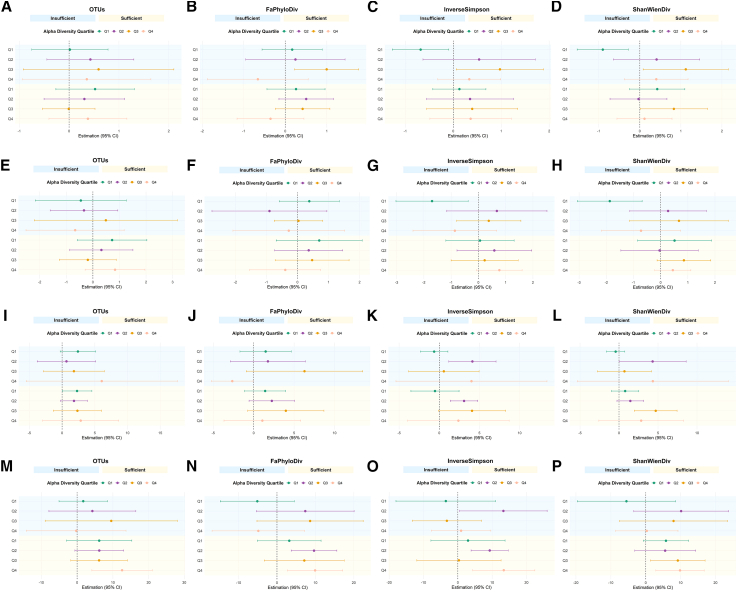
Joint associations of oral microbiome α-diversity and leisure-time physical activity (LTPA) with cognitive function (A–D, E–H, I–L, and M–P) correspond to CERAD immediate recall, CERAD delayed recall, the Animal Fluency Test (AFT), and the Digit Symbol Substitution Test (DSST), respectively. Within each cognitive domain, participants are grouped by quartiles of α-diversity (Observed OTUs, Faith’s phylogenetic diversity, Shannon index, and Inverse Simpson index) and by LTPA category. Points indicate adjusted mean differences in cognitive test scores (with 95% confidence intervals) across α-diversity quartiles and LTPA categories. Background shading highlights LTPA categories, with Insufficient LTPA (<600 MET-min·week^−1^) in light blue and Sufficient LTPA (≥600 MET-min·week^−1^) in light yellow. Data are presented as β with 95% confidence intervals.

In the fully adjusted models (Figure 1), oral microbiome α-diversity partly moderated the associations between LTPA and several cognitive outcomes.

For the CERAD-IR (Table S3), participants with higher Shannon diversity (Q3) who engaged in either insufficient or sufficient LTPA scored significantly higher than the reference group (Q1 with no LTPA), with β = 1.12 (95% CI: 0.083 to 2.157, *p* = 0.036) and β = 0.83 (95% CI: 0.011 to 1.650, *p* = 0.047), respectively. A similar pattern was observed for Inverse Simpson, where the Q3 subgroup with insufficient LTPA performed better than the reference (β = 0.973, 95% CI: 0.064 to 1.881, *p* = 0.037). No clear moderation patterns were observed for CERAD-DR (Table S4), as interaction terms across all α-diversity measures and LTPA categories did not reach statistical significance.

For the Animal Fluency Test (Table S5), significant positive interactions emerged. Participants in the second quartile of Inverse Simpson who engaged in sufficient LTPA had higher AFT scores (β = 3.086, 95% CI: 1.368 to 4.803, *p* = 0.002), and those in Q3 of Shannon diversity showed similar benefits (β = 4.704, 95% CI: 1.960 to 7.448, *p* = 0.002). The p for trend across α-diversity quartiles was significant in both indices (both *p* < 0.01).

For DSST (Table S6), a consistent moderator effect was observed. Participants in the highest quartile of OTUs and sufficient LTPA had significantly greater DSST scores (β = 12.591, 95% CI: 4.135 to 21.047, *p* = 0.007), as did those in the highest quartile of FaPhyloDiv (β = 9.921, 95% CI: 2.742 to 17.099, *p* = 0.011), Shannon diversity (Q4, β = 9.830, 95% CI: 2.893 to 16.766, *p* = 0.009), and Inverse Simpson (Q4, β = 13.389, 95% CI: 4.333 to 22.446, *p* = 0.007). These interaction effects suggest a combined enhancement of cognitive performance when high LTPA participation is associated with greater α-diversity measures.

### Joint associations between β-diversity measures, physical activity, and cognitive function

**Figure 2:**
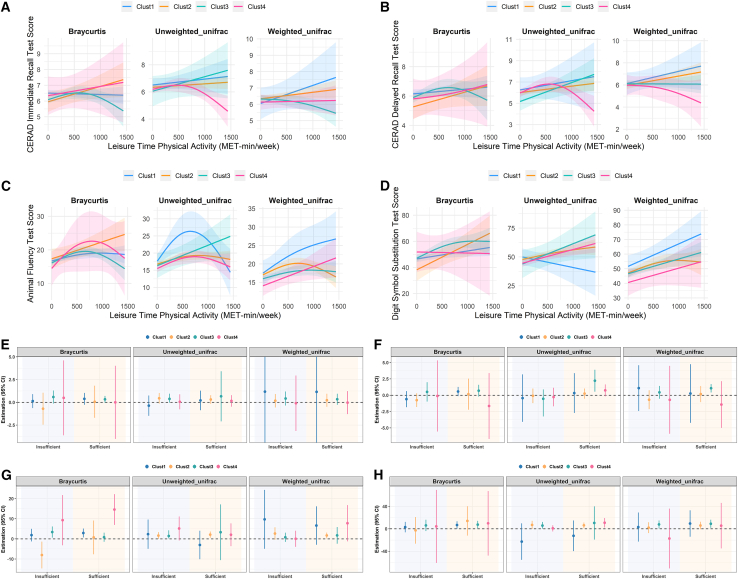
Joint associations of oral microbiome β-diversity and leisure-time physical activity (LTPA) with four domains of cognitive function: CERAD immediate recall, CERAD delayed recall, the Animal Fluency Test (AFT), and the Digit Symbol Substitution Test (DSST) (A–D) Display restricted cubic spline (RCS) models treating LTPA as a continuous exposure, showing cluster-specific, non-linear associations with cognitive scores across three β-diversity metrics: Bray-Curtis, unweighted UniFrac, and weighted UniFrac. (E–H) Present categorical models comparing “Insufficient” (1–600 MET-min/week) and “Sufficient” (>600 MET-min/week) LTPA groups to the “No LTPA” reference group, stratified by β-diversity clusters. Data are presented as β with 95% confidence intervals.

In the restricted cubic-spline (RCS) analyses that modeled LTPA as a continuous exposure (Figure 2), the activity-cognition curves differed markedly by β-diversity cluster. For AFT, scores in Bray-Curtis Cluster 4—and in Cluster 2 under both UniFrac distances—rose steeply until roughly 600–800 MET-min week^−1^ before reaching a plateau, whereas clusters with lower microbial diversity flattened at much lower activity levels. DSST scores increased almost linearly in Bray-Curtis and weighted UniFrac Cluster 3, and in unweighted UniFrac Clusters 2 and 4, up to about 1 000 MET-min week^−1^, after which the slopes attenuated; clusters characterized by lower diversity or distinct composition showed minimal change across the activity range. Spline trajectories for CERAD immediate and delayed recall were largely flat, except for a modest upward bend for unweighted UniFrac Cluster 3 on CERAD-DR, consistent with its small but significant categorical estimate. Taken together, these non-linear patterns indicate cluster-specific thresholds—typically 600–800 MET-min week^−1^ for AFT and 800–1 000 MET-min week^−1^ for DSST—beyond which additional activity yields diminishing returns, underscoring the amplifying role of a diverse oral microbiome on executive function and processing speed.

The RCS findings were mirrored and refined in the fully adjusted categorical models (Figure 2). CERAD-IR (Table S7) showed no consistent moderation, and CERAD-DR (Table S8) displayed only modest effects, limited to unweighted UniFrac Cluster 3 (β = 2.224, 95% CI: 0.544 to 3.904, *p* = 0.029) and weighted UniFrac Cluster 3 (β = 1.069, 95% CI: 0.431 to 1.707, *p* = 0.003).

However, for AFT (Table S9), significant interaction effects emerged across all three β-diversity distance measures. Participants in Bray-Curtis Cluster 4 who engaged in sufficient LTPA scored markedly higher than the reference group (Cluster 1, no LTPA; β = 14.601, 95% CI: 6.965 to 22.236, *p* = 0.014). Comparable benefits were observed for unweighted UniFrac Cluster 2 (β = 2.120, 95% CI: 0.573 to 3.666, *p* = 0.011) and weighted UniFrac Cluster 2 (β = 1.711, 95% CI: 0.276 to 3.146, *p* = 0.023).

Moreover, for DSST (Table S10), LTPA-related gains were most pronounced in Bray-Curtis Cluster 3 (β = 7.527, 95% CI: 0.761 to 14.293, *p* = 0.032), unweighted UniFrac Clusters 2 and 4 (β = 6.252, *p* = 0.018; β = 10.893, *p* = 0.014), and weighted UniFrac Cluster 3 (β = 9.073, 95% CI: 1.863 to 16.284, *p* = 0.017).

### Exploratory analyses of inflammation-related markers

Given the observed associations between LTPA, oral microbiome diversity, and cognitive performance, we further examined inflammation-related indicators to explore potential mechanisms, using white blood cell (WBC) count as an exploratory marker.

In weighted linear regression models, higher continuous LTPA (per 100-MET increase) was associated with lower WBC in both the crude model (β = −0.023, 95% CI −0.038 to −0.007; *p* = 0.004) and the adjusted model (β = −0.025, 95% CI −0.040 to −0.009; *p* = 0.002). When LTPA was categorized, the sufficient activity group (>600 MET-min/week) showed significantly lower WBC compared with inactive participants (crude: β = −0.883, 95% CI −1.552 to −0.214; *p* = 0.013; adjusted: β = −0.923, 95% CI −1.642 to −0.204; *p* = 0.016), whereas the insufficient group (1–600 MET-min/week) was not significantly different from the inactive group (Table S11).

We also evaluated associations between oral microbiome diversity and WBC (Table S12). Among alpha-diversity indices, higher Inverse Simpson diversity was associated with lower WBC in both crude (β = −5.360, 95% CI −9.337 to −1.383; *p* = 0.011) and adjusted models (β = −6.045, 95% CI −10.196 to −1.893; *p* = 0.008). For Shannon-Wiener diversity, the continuous association reached significance in the adjusted model (β = −0.381, 95% CI −0.754 to −0.009; *p* = 0.045), and participants in the highest quartile also had lower WBC (Q4 vs. Q1: β = −0.596, 95% CI −1.155 to −0.038; *p* = 0.039). Other alpha-diversity metrics showed no significant associations. For beta-diversity clustering, unweighted UniFrac clusters were consistently associated with lower WBC compared with Cluster 1 (e.g., Cluster 3 vs. Cluster 1: adjusted β = −2.041, 95% CI −2.622 to −1.460; *p* < 0.001). Moreover, higher WBC was associated with poorer performance on DSST (Table S13), with significant inverse associations in both crude (β = −0.901, 95% CI −1.651 to −0.151; *p* = 0.019) and adjusted models (β = −0.763, 95% CI −1.494 to −0.032; *p* = 0.041).

## Discussion

The present analysis of 575 U.S. adults aged 60–69 years provides three insights. First, LTPA was positively associated with memory, executive function, and processing speed, and achieving guideline-level activity (>600 MET-min week^−1^) corresponded to an ≈ 8-point advantage on the DSST. Second, greater oral microbial α-diversity—especially OTU richness and Inverse-Simpson evenness—was linked to faster processing speed. Third, microbial diversity moderated activity-cognition relations: participants with a favorable oral community structure accrued larger cognitive returns from LTPA. Last but not least, exploratory inflammation-related analyses showed that higher LTPA and greater microbial diversity were associated with lower WBC counts, and higher WBC was in turn related to poorer DSST performance, supporting a potential role of systemic inflammation in this pathway.

Our activity coefficients are larger than the pooled effects observed in the latest longitudinal meta-analysis of 104 cohorts (*n* ≈ 341,000), where each SD increase in baseline activity yielded only a 0.03-SD gain in global cognition and a pooled risk ratio of 0.97 for incident impairment.^9^ Several factors may explain this discrepancy: (i) we focused on adults entering the “hazard window” for cognitive decline (60–69 years), a stage at which activity might exert maximal benefit; (ii) DSST is more sensitive than the global composites used in many cohort studies; and (iii) our survey-weighted modeling reduced exposure misclassification by preserving the continuous MET gradient. At the same time, the per-100-MET-min coefficients for memory outcomes are numerically small—for example, a 0.013-point increase in CERAD-IR (range 0–30) for each additional 100 MET-min·week^−1^, which corresponds to roughly 25 min of moderate walking per week. When we compare inactive participants with those achieving ≥600 MET-min·week^−1^, the between-group differences in DSST (≈8 points) and CERAD-DR (≈0.55 points) fall within the range reported in other cohort and intervention studies and may be meaningful at the population level. Nevertheless, our findings align with RCT evidence showing small-to-moderate benefits of aerobic and resistance training across cognitive domains (SMD ≈ 0.2–0.3).^27^^,^^28^

Independent of LTPA, participants in the upper-diversity quartiles exhibited faster processing speed, echoing a recent study in which higher Shannon and Simpson indices predicted superior executive function and subjective memory.^29^ However, compared with the previous study mentioned,^29^ our study extends the field in several important respects. Lin et al. restricted their outcomes to DSST and a dichotomized self-reported memory measure, which limited sensitivity to graded and non-linear relationships. In contrast, we evaluated four validated cognitive domains as continuous outcomes and explicitly modeled potential non-linearity using restricted cubic splines. Moreover, whereas previous literature focused on a single α-diversity index (Observed OTUs),^29^ we incorporated four widely used indices—Observed OTUs/ASVs, Faith’s phylogenetic diversity, Shannon, and Inverse Simpson—thereby reducing dependence on any one metric. For β-diversity, our analysis also moved beyond their sole use of unweighted UniFrac by applying multiple dissimilarity measures (Bray-Curtis, weighted and unweighted UniFrac) and *a priori* clustering to better capture heterogeneity in microbial community structure. Together, these methodological advances provide a more robust characterization of how oral microbiome diversity relates to cognition. Taken together, the literature and our results suggest that maintaining a diverse oral ecosystem may safeguard cognitive processing speed, a domain that not only plays a central role in daily functioning among older adults, but whose decline is also associated with diminished real-world functional capacity.^30^

The amplification of activity benefits among diversity-rich microbiomes advances the emerging concept of a bi-directional oral-brain-exercise axis. Experimental work demonstrates that 16 weeks of moderate-intensity continuous training increases oral Shannon diversity and reduces pathogenic taxa in older adults.^25^ Mechanistically, exercise elevates brain-derived neurotrophic factor and improves cerebral perfusion,^31^ whereas oral dysbiosis fuels systemic inflammation and blood-brain-barrier disruption.^32^ A diverse, commensal-dominated oral microbiome may temper inflammatory signaling,^33^ thereby lowering the physiological “noise floor” against which the neuroplastic benefits of exercise can manifest.^34^ Conversely, dysbiosis could blunt exercise-induced gains by maintaining a pro-inflammatory milieu. Our moderation analyses lend epidemiological support to this hypothesis, revealing that the cognitive pay-off from physical activity is contingent on microbial context. However, these biological links are still incompletely understood; our data show the statistical modification of the LTPA-cognition association by oral diversity, but they do not establish a direct joint causal pathway, which will need to be tested in longitudinal and experimental studies.

In summary, in this cross-sectional, nationally representative analysis of U.S. older adults, higher levels of leisure-time physical activity were positively associated with multiple cognitive domains, most notably processing speed, while greater oral microbial α-diversity showed a similar positive association with cognitive performance. Moreover, the strength of the activity-cognition association varied according to oral microbiome profile: individuals with more diverse, commensal-rich communities exhibited stronger correlations between physical activity and cognition than those with lower diversity. These findings highlight two modifiable characteristics—habitual physical activity and oral microbial composition—that appear to co-vary with late-life cognitive function and with each other. Because the data are observational, causal inferences cannot be drawn; nevertheless, the patterns underline the importance of considering both behavioral and biological contexts when examining cognitive aging.

### Limitations of the study

Strengths include the nationally representative design, high-throughput 16S rRNA profiling of oral rinse samples, concurrent assessment of four cognitive domains, careful control for demographic, behavioral, and health-related confounders, and the use of both categorical and continuous LTPA indicators together with spline-based and interaction models. Meanwhile, several limitations warrant caution. First, the cross-sectional design precludes causal inference; it is also possible that older adults with early subtle cognitive problems already move less and pay less attention to oral care, which could in turn influence the oral microbiome. Longitudinal cohorts with repeated assessments of activity, oral microbiota, and cognition will be needed to clarify directionality. Second, LTPA was self-reported, introducing recall bias. At the same time, the proportion of participants reporting no LTPA in our sample is similar to national surveillance data, where about 55–60% of U.S. adults aged 60 years and older report no LTPA.^35^^,^^36^ Third, only participants with complete data on oral microbiome, LTPA, cognition, and covariates were included; some selection bias is possible even though survey weights for the oral microbiome subsample were applied. Finally, we examined several related activity metrics, cognitive outcomes, diversity measures, and interaction terms. Our main conclusions are based on patterns that are consistent across models, but some individual *p* values—especially those close to 0.05—should be interpreted cautiously and verified in other datasets where possible.

Despite these limitations, the data highlight two modifiable factors—physical activity and oral microbial diversity—that converge on shared inflammatory and neurovascular pathways. Publicly available datasets that jointly include oral 16S profiles, LTPA, and cognitive outcomes remain scarce, which explains why the literature in this area is still thin. Within NHANES, for example, oral microbiome sequencing was conducted only in 2009–2012 cycles, whereas cognitive testing was implemented in 2011–2014 cycles, leaving only a narrow temporal overlap. If a fully independent external cohort becomes accessible, we would readily incorporate it, but at present, the options are very limited. Even so, our findings align closely with WHO guidelines (≥600 MET-min week^−1^), suggesting that standard activity prescriptions may already approximate the optimal dose when coupled with good oral health. Clinically, integrating periodontal care or probiotic interventions with exercise programs could yield additive cognitive benefits. This perspective is further supported by a recent study, which reported that among 2,059 older adults, engaging in multi-domain lifestyle behaviors—including physical activity, social engagement, and cognitive stimulation—significantly slowed cognitive decline. Notably, these effects remained robust even after adjusting for underlying neuropathology, suggesting that integrated behavioral-biological interventions may confer additional cognitive benefits.^37^^,^^38^ Considering that a single oral-rinse sample may not capture temporal microbiome dynamics, and 16S profiling cannot resolve species- or strain-level neuroactive pathways, device-based activity tracking and multi-omics profiling will further clarify directionality and mechanistic mediators.

## Resource availability

### Lead contact

Further information and requests for resources, data, and code should be directed to and will be fulfilled by the lead contact, Xindong Ma (maxd@mail.tsinghua.edu.cn).

### Materials availability

This study did not generate any new unique reagents, cell lines, or animal models.

### Data and code availability

• This article analyzes existing, publicly available data from the NHANES 2011–2012 survey, including the oral microbiome subsample, which can be downloaded from the National Center for Health Statistics website.
• The filtered analytic dataset used for all analyses has been deposited as Data S1 at Zenodo (https://zenodo.org/records/18616290). This article does not report new sequencing data or other newly generated datasets.
• The analysis codes have been deposited as Data S2 at Zenodo (https://zenodo.org/records/18616313). All R scripts and analysis code used in this study are available from the lead contact upon reasonable request.
• Any additional information required to reanalyze the data reported in this article is available from the lead contact upon request.

## Acknowledgments

This study was supported by the Institute of Sports Development Research of Tsinghua University (Research on John Mo’s thought and practice of Physical Education), the Tsinghua University Initiative Scientific Research Program (2024THZWYY05), the Guangzhou Concord Medical Humanities Research and Education Fund (23000-3050070), and the Tsinghua University Computational Social Science Research Program (202501020008).

## Author contributions

Y. Y.: conceptualization, methodology, formal analysis, validation, investigation, writing – original draft, writing – review and editing; Y. C.: writing – original draft, visualization, validation, and writing – review and editing; K. Z.: writing – original draft, visualization, validation, and writing – review and editing; H. Y.: methodology, investigation, and writing – review and editing; C. Y.: methodology and investigation; Q. W.: validation and writing – review and editing; Y. C.: writing – review and editing, supervision, and project administration; X. M.: conceptualization, supervision, project administration, and funding acquisition. All authors read and approved the final article.

## Declaration of interests

The authors declare no competing interests.

## Declaration of generative AI and AI-assisted technologies in the writing process

During the preparation of this work, the authors used no Generative AI and AI-assisted technologies in the writing process and take full responsibility for the content of the publication.

## STAR★Methods

### Key resources table

**Table:** 

REAGENT or RESOURCE	SOURCE	IDENTIFIER
**Deposited data**		

NHANES 2011–2012 oral microbiome and cognitive data	National Health and Nutrition Examination Survey (NHANES), National Center for Health Statistics	NHANES 2011–2012; Oral Microbiome Project 2009–2012 (public-use datasets) (Filtered NHANES 2011–2012 analytic dataset used for all analyses in this study can be found at https://zenodo.org/records/18616290)

**Software and algorithms**		

R, version 4.3.2	R Foundation for Statistical Computing	https://www.r-project.org (R scripts and session information used to reproduce the analyses and figures can be found at https://zenodo.org/records/18616313)
QIIME 2, version 2023.9	QIIME 2 Development Team	https://qiime2.org

### Experimental model and study participant details

#### Study population

NHANES is a continuous, cross-sectional program that employs a complex, multistage probability design to collect nationally representative health data from the non-institutionalized U.S. population. We confined our analysis to the 2011–2012 cycle—the only examination waves in which the oral-rinse 16S rRNA sequencing carried out for the NHANES Oral Microbiome Project (available 2009–2012) coincided with the full battery of cognitive tests (administered 2011–2014). This overlap allowed oral microbial profiles and cognitive outcomes to be measured simultaneously.

From the 9,708 individuals examined in 2011–2012, we limited the sample to adults aged 60–69 y because all four cognitive tests were administered solely within this age band. After excluding participants less than 60 years (n = 7,917), there were 1,791 participants left. Of the 1,791 participants, we excluded those lacking oral-rinse specimen’s assessment (n = 813), missing any cognitive assessment (n = 196), leisure-time physical-activity data (n = 115), or missing essential covariate information for multivariable adjustment (n = 92), yielding a final analytic cohort of 575 older adults. Mobile Examination Centre (MEC) sampling weights specific to the oral-microbiome subsample were applied to preserve national representativeness. Thus, our estimates pertain to community-dwelling adults aged 60–69 years with complete oral microbiome, LTPA, and cognitive data.

The NHANES protocol was approved by the National Center for Health Statistics Research Ethics Review Board, and written informed consent was obtained from all participants. Because the data are publicly available and fully de-identified, no additional institutional review was required.

### Method details

#### Exposure variable: Leisure-time physical activity

LTPA was quantified from the NHANES Physical Activity Questionnaire, which elicits the frequency (sessions week^-1^) and average duration (minutes session^-1^) of moderate- and vigorous-intensity sports, fitness, and recreational pursuits undertaken during the preceding 30 days.^39^^,^^40^ Participants are first asked whether they engage in any such activity lasting at least 10 minutes at a time; only bouts of ≥10 minutes are recorded and used to derive volume. Occupational, transportation, and household activities were not included, in line with prior cognitive-epidemiology work that focuses on volitional movement. For each respondent, weekly minutes of moderate activity were multiplied by a standard intensity of 4.0 METs, whereas vigorous minutes were assigned 8.0 METs; minutes of vigorous activity were also doubled before aggregation to reflect the “2 min moderate = 1 min vigorous” weighting recommended by the 2018 Physical Activity Guidelines for Americans and earlier compendia.^41^ The products were summed to obtain total MET-minutes week^-1^, which were subsequently rescaled in units of 100 MET-minutes week^-1^ to improve model convergence.

Consistent with previous population-based studies,^42^^,^^43^ LTPA was analyzed both continuously and categorically: participants who reported no qualifying leisure-time sport, fitness, or recreational activity in the past 30 days (0 MET-minutes·week^-1^) formed the “none” group; those accumulating 1–<600 MET-minutes week^-1^ were classified as “insufficient”; and those achieving ≥600 MET-minutes week^-1^ constituted the “sufficient” group, corresponding to at least 150 min week^-1^ of moderate-intensity or 75 min week^-1^ of vigorous-intensity exercise.^44^

#### Exposure variable: Oral microbiome

NHANES collected an oral-rinse sample from every participant in 2011–2012 year-cycle. At the mobile examination center, each person rinsed with a commercial mouth-wash for 5 s, gargled three more times for 5 s each, and expelled the fluid into a sterile tube.^45^ Genomic DNA was purified with the Puregene® kit together with blank tubes and two mock bacterial communities that served as quality controls. All plates were stored at –80 °C and shipped on dry ice to the Knight Laboratory (University of California, San Diego) for 16S rRNA gene analysis. The V4 region was amplified with the 515F/806R primer pair and sequenced on an Illumina HiSeq 2500 platform (2 × 125 bp) following the protocol described by Caporaso et al.^46^

Raw reads were demultiplexed, trimmed, and error-corrected in QIIME 2 (v 2023.9). Exact sequence variants (amplicon sequence variants, ASVs) were inferred with DADA2, and taxonomy was assigned against the SILVA-138 database.^47^ Negative controls produced negligible read counts and were therefore discarded. To ensure comparability across participants, each library was randomly subsampled to 10,000 reads.

Two sets of microbiome features were carried forward as exposures. First, four within-sample (α) diversity indices—OTU richness, Faith’s phylogenetic diversity, Shannon index, and Inverse Simpson index—were analyzed as continuous variables. Second, between-sample (β) diversity was summarized by Bray–Curtis dissimilarity and by unweighted and weighted UniFrac distances. These cluster labels were treated as categorical variables in the regression models, with Cluster 1 (lowest diversity) serving as the reference group.^48^ Moreover, for subgroup analyses based on oral microbiome diversity, all participants in the analytic sample had complete oral microbiome sequencing data. Alpha-diversity were categorized into quartiles, with the following sample sizes: Observed OTUs (Q1 n = 144; Q2 n = 143; Q3 n = 144; Q4 n = 144), Shannon–Wiener diversity (Q1 n = 143; Q2 n = 141; Q3 n = 146; Q4 n = 145), Faith’s phylogenetic diversity (Q1 n = 143; Q2 n = 144; Q3 n = 143; Q4 n = 145), and Inverse Simpson diversity (Q1 n = 138; Q2 n = 130; Q3 n = 126; Q4 n = 181).

#### Outcome variable: Cognitive function

Cognitive performance was evaluated with the four tests administered in NHANES 2011–2012 to participants aged 60–69 y. Testing was conducted in a quiet room of the mobile examination center by certified examiners who followed a standardized script provided by the National Center for Health Statistics. Episodic memory was captured with the Consortium to Establish a Registry for Alzheimer’s Disease (CERAD) Word Learning sub-test: participants attempted to learn ten concrete nouns across three immediate-recall trials (score range 0–30) and reproduced the list again after a 10-min delay (range 0–10).^49^ Semantic verbal fluency was assessed with the Animal Fluency Test, which required respondents to name as many different animals as possible within 60 s; the total number of non-repeated, correct animals constituted the score, with typical values between 0 and 40.^50^ Processing speed and executive control were measured with the Digit Symbol Substitution Test from the Wechsler Adult Intelligence Scale, third edition; during a 120 s interval, examinees matched symbols to digits according to a reference key printed at the top of the page, and the count of correctly transcribed pairs (possible range 0–133) formed the outcome.^51^ Raw scores from each test were analyzed as continuous variables in regression models, with higher values indicating better cognitive function.

#### Covariates

Potential confounders were selected *a priori* on the basis of established links with both oral microbiota and late-life cognition. Age, sex (male/female) and self-reported race/ethnicity (non-Hispanic White, non-Hispanic Black, Mexican American, other) were recorded during the household interview, while standing height and weight were measured at the mobile examination center and used to calculate body-mass index (BMI, kg m^-2^). BMI entered the models as a three-level variable (< 25, 25–<30, ≥ 30 kg m^-2^), reflecting normal weight, overweight and obesity. Socio-economic position was captured by marital status (married/living with partner vs. separated/divorced/widowed/never married), education (less than high school, high-school graduate, college or above) and the poverty-income ratio, which expresses household income relative to the federal poverty line and was grouped as < 1.0, 1.0–< 3.0 or ≥ 3.0. Following previous studies,^52^^,^^53^ health-behavior covariates consisted of cigarette-smoking status—never, former or current—and alcohol-use status, classified as none, moderate (≤ 1 drink d^-1^ for women or ≤ 2 drinks d^-1^ for men) or high (> 1 or > 2 drinks d^-1^, respectively). Finally, three self-reported physician diagnoses were included as binary indicators of chronic disease burden: diabetes mellitus, hypertension and cardiovascular disease (any history of myocardial infarction, angina or stroke).

Moreover, white blood cell (WBC) count was included as an exploratory inflammation-related marker. In NHANES, WBC was obtained from the complete blood count performed on whole blood collected in EDTA tubes during the mobile examination center visit and analyzed using an automated hematology analyzer (Coulter DxH 800; Beckman Coulter). In the present study, WBC count (10^3^ cells/μL) was incorporated as a continuous variable in regression models to evaluate its associations with LTPA and cognitive outcomes, and to support mechanistic rationale via systemic inflammation.

### Quantification and statistical analysis

All computations were performed in R (version 4.3.2; R Foundation for Statistical Computing, Vienna, Austria). To preserve the complex, multistage sampling structure of NHANES, we incorporated the mobile-examination-centre (MEC) subsample weights that are specific to the oral-microbiome dataset, together with strata and primary sampling units, by means of the survey package. Descriptive statistics were expressed as weighted means ± standard deviations for continuous variables and weighted percentages for categorical variables.

Primary associations between leisure-time physical activity (LTPA) and each cognitive test score were estimated with survey-weighted multivariable linear-regression models that progressively adjusted for the covariates described in Section 4.5. LTPA entered the models both as a continuous term, scaled in 100 MET-min week^-1^, and as a three-level guideline variable (none, insufficient < 600, sufficient ≥ 600 MET-min week^-1^). To facilitate comparison with these behavioral exposures, α-diversity indices were analyzed per one-quartile increase, whereas β-diversity was represented by the four-level community-structure clusters derived from principal-coordinate axes and k-means partitioning. Because the relation between continuous LTPA and cognition could be non-linear, we fitted restricted cubic-spline (RCS) functions with three knots placed at the 10th, 50th and 90th weighted percentiles of LTPA. Separate RCS curves were drawn within each β-diversity cluster, allowing the visualization of cluster-specific dose–response patterns. Two-sided p < 0.05 denoted statistical significance.

## Supplemental information

Document S1. Tables S1–S13

Data S1. Filtered NHANES 2011–2012 analytic dataset used for all analyses in this study

Data S2. R scripts and session information used to reproduce the analyses and figures
