# Supplementary material for: Oral microbiome diversity shapes the association between leisure-time physical activity and cognitive function among older adults
Source: iScience. 2026 Feb 19;29(3):115086. doi: 10.1016/j.isci.2026.115086 (PMC12989845; doi:10.1016/j.isci.2026.115086)
Supplement: Document S1. Tables S1–S13 [file mmc1.pdf]

iScience, Volume *29*

Supplemental information

Oral microbiome diversity shapes the association between leisure-time physical activity and cognitive function among older adults

Yanwei You, Yicong Cui, Kefeng Zheng, Haopeng Yang, Chuanwen Yu, Qingyuan Wang, Yuquan Chen, and Xindong Ma

Table S1. Association results of leisure time physical activity, four alpha-diversity metrics, three beta-diversity measures and CERAD-IR using weighted linear regression models

		Crude model		Adjusted Model	
		β (95% CI)	*P-value*	β (95% CI)	*P-value*
Leisure-time physical activity	Continuous (Per 100-MET increase)	0.01(0.001,0.019)	0.039	0.013(0.002, 0.023)	0.019
	No	Reference		Reference	
	Insufficient (1-600 MET)	0.267(-0.266, 0.800)	0.291	0.314(-0.277,0.905)	0.275
	Sufficient (> 600 MET)	0.283(-0.160, 0.725)	0.185	0.29(-0.136,0.717)	0.167
OTUs	Continuous	-0.001(-0.007,0.005)	0.643	-0.002(-0.008,0.004)	0.437
	Q1 (<80.20)	Reference		Reference	
	Q2 (80.20-101.08)	0.17(-0.247,0.588)	0.391	0.169(-0.284,0.623)	0.437
	Q3 (101.08-126.71)	-0.011(-0.364,0.342)	0.945	-0.069(-0.414,0.276)	0.675
	Q4 (>126.71)	-0.021(-0.659,0.618)	0.946	-0.087(-0.677,0.503)	0.756
FaPhyloDiv	Continuous	-0.029(-0.104,0.046)	0.426	-0.02(-0.097,0.056)	0.579
	Q1 (<10.58)	Reference		Reference	
	Q2 (10.58-12.32)	-0.231(-0.734,0.273)	0.343	-0.22(-0.728,0.288)	0.365
	Q3 (12.32-14.34)	0.007(-0.554,0.568)	0.980	0.136(-0.415,0.687)	0.601
	Q4 (>14.34)	-0.342(-0.830,0.146)	0.155	-0.282(-0.782,0.218)	0.243
InvSimpson	Continuous	-0.286(-3.728,3.155)	0.862	0.127(-3.409,3.663)	0.940
	Q1 (<0.88)	Reference		Reference	
	Q2 (0.88-0.91)	0.143(-0.308,0.594)	0.507	0.205(-0.188,0.598)	0.277
	Q3 (0.91-0.93)	0.015(-0.548,0.578)	0.955	0.07(-0.476,0.616)	0.784
	Q4 (>0.93)	0.314(-0.319,0.947)	0.305	0.335(-0.308,0.978)	0.279

ShanWienDiv	Continuous	0.005(-0.305,0.314)	0.974	0.028(-0.293,0.350)	0.853
	Q1 (<4.09)	Reference		Reference	
	Q2 (4.09-4.49)	-0.316(-0.802,0.170)	0.185	-0.282(-0.742,0.177)	0.205
	Q3 (4.49-4.93)	-0.169(-0.697,0.358)	0.502	-0.169(-0.723,0.384)	0.518
	Q4 (>4.93)	-0.027(-0.545,0.491)	0.912	0.005(-0.531,0.541)	0.985
Braycurtis distance	Cluster 1	Reference		Reference	
	Cluster 2	-0.523(-1.438,0.392)	0.237	-0.183(-0.774, 0.408)	0.518
	Cluster 3	-0.591(-1.216,0.033)	0.061	-0.431(-0.829,-0.034)	0.035
	Cluster 4	-0.883(-2.297,0.532)	0.199	-0.429(-1.415, 0.558)	0.367
Unweighed Unifrac	Cluster 1	Reference		Reference	
	Cluster 2	0.179(-0.459,0.816)	0.553	0.209(-0.475,0.893)	0.524
	Cluster 3	0.422(-0.832,1.676)	0.477	0.55(-0.499,1.599)	0.280
	Cluster 4	0.09(-0.577,0.757)	0.773	0.007(-0.718,0.733)	0.983
Weighed Unifrac	Cluster 1	Reference		Reference	
	Cluster 2	0.445(-0.198,1.088)	0.158	0.387(-0.185,0.958)	0.169
	Cluster 3	0.292(-0.388,0.973)	0.368	0.271(-0.326,0.869)	0.371
	Cluster 4	0.205(-0.342,0.751)	0.431	0.064(-0.310,0.437)	0.720

Notes: Crude model, no covariates were adjusted. Adjusted Model, age, sex, race/ethnicity, body mass index, marital status, education, poverty income ratio, smoke status, alcohol use status, and chronic diseases were adjusted.

Table S2. Association results of leisure time physical activity, four alpha-diversity metrics, three beta-diversity measures and CERAD-DR using weighted linear regression models

		Crude model		Adjusted Model	
		β (95% CI)	*P-value*	β (95% CI)	*P-value*
Leisure-time physical activity	Continuous (Per 100-MET increase)	0.014(0.003,0.024)	0.016	0.023(0.010,0.036)	0.002
	No	Reference		Reference	
	Insufficient (1-600 MET)	-0.122(-1.106,0.861)	0.794	-0.259(-1.046,0.527)	0.487
	Sufficient (> 600 MET)	0.443(-0.159,1.045)	0.136	0.548(0.033,1.062)	0.039
OTUs	Continuous	-0.001(-0.010,0.009)	0.894	-0.002(-0.012,0.007)	0.628
	Q1 (<80.20)	Reference		Reference	
	Q2 (80.20-101.08)	0.22(-0.577,1.016)	0.559	0.217(-0.574,1.007)	0.566
	Q3 (101.08-126.71)	0.1(-0.611,0.810)	0.765	-0.015(-0.650,0.620)	0.960
	Q4 (>126.71)	0.017(-0.908,0.943)	0.968	-0.114(-1.019,0.792)	0.792
FaPhyloDiv	Continuous	-0.01(-0.108,0.088)	0.830	-0.027(-0.121,0.067)	0.552
	Q1 (<10.58)	Reference		Reference	
	Q2 (10.58-12.32)	0.008(-0.512,0.528)	0.974	-0.011(-0.552,0.529)	0.965
	Q3 (12.32-14.34)	0.337(-0.457,1.130)	0.374	0.09(-0.661,0.841)	0.801
	Q4 (>14.34)	-0.171(-0.901,0.559)	0.619	-0.287(-0.990,0.416)	0.395
InvSimpson	Continuous	1.98(-3.948,7.908)	0.486	1.167(-4.669,7.003)	0.677
	Q1 (<0.88)	Reference		Reference	
	Q2 (0.88-0.91)	0.273(-0.614,1.159)	0.515	0.158(-0.771,1.087)	0.721
	Q3 (0.91-0.93)	0.285(-0.391,0.961)	0.377	0.183(-0.503,0.870)	0.576
	Q4 (>0.93)	0.768(-0.203,1.738)	0.110	0.725(-0.174,1.624)	0.106

ShanWienDiv	Continuous	0.186(-0.342,0.714)	0.462	0.14(-0.358,0.637)	0.560
	Q1 (<4.09)	Reference		Reference	
	Q2 (4.09-4.49)	-0.131(-0.862,0.601)	0.740	-0.196(-0.905,0.514)	0.563
	Q3 (4.49-4.93)	0.184(-0.761,1.128)	0.680	0.182(-0.648,1.012)	0.645
	Q4 (>4.93)	0.339(-0.575,1.253)	0.435	0.27(-0.659,1.199)	0.543
Braycurtis distance	Cluster 1	Reference		Reference	
	Cluster 2	-0.523(-1.438,0.392)	0.237	-0.926(-1.894,0.041)	0.059
	Cluster 3	-0.591(-1.216,0.033)	0.061	-0.623(-1.280,0.034)	0.061
	Cluster 4	-0.883(-2.297,0.532)	0.199	-0.539(-2.042,0.963)	0.454
Unweighed Unifrac	Cluster 1	Reference		Reference	
	Cluster 2	0.207(-1.237,1.651)	0.760	0.274(-1.164,1.712)	0.689
	Cluster 3	-0.361(-2.876,2.153)	0.760	-0.094(-1.979,1.791)	0.916
	Cluster 4	0.106(-1.417,1.628)	0.882	-0.051(-1.498,1.397)	0.941
Weighed Unifrac	Cluster 1	Reference		Reference	
	Cluster 2	0.26(-1.114,1.634)	0.689	0.127(-1.160,1.413)	0.836
	Cluster 3	0.368(-0.716,1.452)	0.476	0.306(-0.720,1.331)	0.533
	Cluster 4	-0.236(-1.784,1.311)	0.747	-0.416(-1.974,1.142)	0.576

Notes: Crude model, no covariates were adjusted. Adjusted Model, age, sex, race/ethnicity, body mass index, marital status, education, poverty income ratio, smoke status, alcohol use status, and chronic diseases were adjusted.

Table S3. Moderator effect of four α-diversity measures on leisure-time physical activity and CERAD-IR using fully adjusted weighted linear regression models.

	No Leisure-time PA	Insufficient Leisure-time PA	p	Sufficient Leisure-time PA	p	p for trend
OTUs						
Q1	Reference	0.016(-0.749,0.782)	0.964	0.522(-0.265,1.309)	0.178	0.175
Q2	Reference	0.428(-0.445,1.300)	0.313	0.307(-0.502,1.116)	0.431	0.413
Q3	Reference	0.592(-0.918,2.103)	0.416	-0.007(-0.534,0.521)	0.978	0.95
Q4	Reference	0.356(-0.930,1.642)	0.560	0.378(-0.404,1.160)	0.316	0.318
FaPhyloDiv						
Q1	Reference	0.167(-0.559,0.892)	0.630	0.259(-0.441,0.958)	0.441	0.433
Q2	Reference	0.245(-0.951,1.441)	0.668	0.504(-0.152,1.161)	0.122	0.121
Q3	Reference	0.997(0.225,1.769)	0.015	0.418(-0.239,1.076)	0.195	0.212
Q4	Reference	-0.66(-1.872,0.552)	0.260	-0.355(-1.161,0.451)	0.359	0.343
ShanWienDiv						
Q1	Reference	-0.901(-1.525,-0.278)	0.008	0.425(-0.249, 1.099)	0.198	0.231
Q2	Reference	0.409(-0.642,1.460)	0.420	-0.031(-0.725,0.662)	0.924	0.908
Q3	Reference	1.12(0.083,2.157)	0.036	0.83(0.011,1.650)	0.047	0.044
Q4	Reference	0.402(-0.373,1.176)	0.285	0.115(-0.556,0.785)	0.719	0.693
InverseSimpson						
Q1	Reference	-0.68(-1.269,-0.092)	0.027	0.128(-0.426, 0.681)	0.627	0.667
Q2	Reference	0.539(-0.631,1.710)	0.340	0.35(-0.555,1.256)	0.420	0.426
Q3	Reference	0.973(0.064,1.881)	0.037	0.395(-0.554,1.345)	0.389	0.379
Q4	Reference	0.334(-0.324,0.991)	0.295	0.362(-0.488,1.213)	0.376	0.37

Table S4. Moderator effect of four α-diversity measures on leisure-time physical activity and CERAD-DR using fully adjusted weighted linear regression models.

	No Leisure-time PA	Insufficient Leisure-time PA	p	Sufficient Leisure-time PA	p	p for trend
OTUs						
Q1	Reference	-0.453(-2.165,1.258)	0.581	0.724(-0.586,2.033)	0.257	0.265
Q2	Reference	-0.335(-1.607,0.937)	0.582	0.32(-0.869,1.508)	0.575	0.587
Q3	Reference	0.488(-2.202,3.177)	0.705	-0.19(-1.260,0.880)	0.710	0.698
Q4	Reference	-0.668(-2.516,1.179)	0.448	0.834(-0.292,1.960)	0.133	0.146
FaPhyloDiv						
Q1	Reference	0.38(-0.588,1.347)	0.414	0.7(-0.685,2.085)	0.297	0.293
Q2	Reference	-0.903(-2.754,0.947)	0.315	0.361(-0.736,1.458)	0.494	0.53
Q3	Reference	0.033(-0.748,0.814)	0.929	0.472(-0.719,1.663)	0.412	0.408
Q4	Reference	-0.281(-2.079,1.516)	0.741	-0.394(-1.535,0.746)	0.469	0.465
ShanWienDiv						
Q1	Reference	-1.873(-3.066,-0.680)	0.005	0.512(-0.850, 1.875)	0.433	0.477
Q2	Reference	0.274(-1.137,1.684)	0.685	-0.04(-1.465,1.385)	0.953	0.947
Q3	Reference	0.674(-1.144,2.492)	0.440	0.856(-0.131,1.844)	0.084	0.083
Q4	Reference	-0.723(-2.178,0.733)	0.305	0.449(-0.217,1.115)	0.170	0.215
InverseSimpson						
Q1	Reference	-1.7(-3.024,-0.375)	0.016	0.06(-1.192, 1.311)	0.920	0.973
Q2	Reference	0.678(-1.159,2.516)	0.442	0.585(-0.785,1.955)	0.375	0.377
Q3	Reference	0.381(-0.798,1.559)	0.502	0.23(-1.002,1.461)	0.697	0.695
Q4	Reference	-0.864(-2.398,0.670)	0.247	0.769(-0.065,1.604)	0.068	0.061

Table S5. Moderator effect of four α-diversity measures on leisure-time physical activity and AFT using fully adjusted weighted linear regression models.

	No Leisure-time PA	Insufficient Leisure-time PA	p	Sufficient Leisure-time PA	p	p for trend
OTUs						
Q1	Reference	2.417(-0.286,5.120)	0.076	2.292(0.029,4.554)	0.047	0.038
Q2	Reference	0.668(-3.792,5.127)	0.754	1.818(-0.233,3.868)	0.078	0.08
Q3	Reference	1.809(-2.841,6.458)	0.420	2.342(-1.325,6.009)	0.193	0.191
Q4	Reference	6.064(-5.490,17.618)	0.277	2.845(-2.968, 8.658)	0.310	0.314
FaPhyloDiv						
Q1	Reference	1.482(-1.695,4.658)	0.334	1.421(-1.097,3.938)	0.246	0.229
Q2	Reference	1.761(-2.886,6.408)	0.432	2.246(-0.569,5.061)	0.110	0.11
Q3	Reference	6.286(-0.914,13.486)	0.082	3.98(-0.735, 8.696)	0.092	0.084
Q4	Reference	-2.645(-5.213,-0.077)	0.044	1.075(-3.671, 5.820)	0.633	0.673
ShanWienDiv						
Q1	Reference	-0.434(-1.605,0.737)	0.440	0.785(-0.960,2.531)	0.351	0.363
Q2	Reference	4.309(0.007,8.611)	0.050	1.448(-0.264,3.160)	0.092	0.109
Q3	Reference	0.696(-2.767,4.159)	0.673	4.704(1.960,7.448)	0.002	0.003
Q4	Reference	4.337(-5.344,14.018)	0.353	2.837(-2.588, 8.262)	0.281	0.287
InverseSimpson						
Q1	Reference	-0.637(-2.352,1.079)	0.437	-0.533(-3.528,2.463)	0.707	0.7
Q2	Reference	4.124(1.180,7.069)	0.009	3.086(1.368,4.803)	0.002	0.001
Q3	Reference	0.567(-3.824,4.957)	0.787	4.081(-0.101,8.263)	0.055	0.055
Q4	Reference	4.009(-5.394,13.411)	0.376	2.377(-3.939, 8.694)	0.433	0.429

Table S6. Moderator effect of four α-diversity measures on leisure-time physical activity and DSST using fully adjusted weighted linear regression models.

	No Leisure-time PA	Insufficient Leisure-time PA	p	Sufficient Leisure-time PA	p	p for trend
OTUs						
Q1	Reference	1.715(-5.059, 8.488)	0.597	6.182(-2.946,15.309)	0.169	0.172
Q2	Reference	4.272(-7.885,16.428)	0.465	6.205(-0.624,13.034)	0.072	0.071
Q3	Reference	9.553(-8.944,28.050)	0.288	6.15(-1.775,14.075)	0.119	0.118
Q4	Reference	-0.253(-14.202,13.697)	0.969	12.591(4.135,21.047)	0.007	0.008
FaPhyloDiv						
Q1	Reference	-5.117(-14.769, 4.536)	0.275	3.199(-5.078,11.476)	0.421	0.443
Q2	Reference	7.36(-5.368,20.088)	0.237	9.644(3.707,15.581)	0.003	0.004
Q3	Reference	8.623(-5.209,22.454)	0.204	7.111(-3.303,17.525)	0.166	0.156
Q4	Reference	-4.86(-16.900, 7.179)	0.399	9.921(2.742,17.099)	0.011	0.017
ShanWienDiv						
Q1	Reference	-5.515(-19.587, 8.558)	0.415	5.808(-0.659,12.276)	0.075	0.081
Q2	Reference	10.151(-3.433,23.735)	0.132	5.577(-3.147,14.302)	0.193	0.186
Q3	Reference	7.982(-7.492,23.455)	0.287	9.203(1.383,17.023)	0.024	0.024
Q4	Reference	0.313(-8.637, 9.262)	0.941	9.83(2.893,16.766)	0.009	0.01
InverseSimpson						
Q1	Reference	-3.498(-18.029,11.032)	0.612	2.967(-7.836,13.770)	0.563	0.576
Q2	Reference	13.271(0.516,26.025)	0.042	9.285(3.862,14.709)	0.003	0.002
Q3	Reference	-3.202(-13.289, 6.885)	0.509	0.321(-11.964,12.606)	0.956	0.958
Q4	Reference	0.937(-7.758, 9.633)	0.820	13.389(4.333,22.446)	0.007	0.008

Table S7. Moderator effect of three β-diversity measures on leisure-time physical activity and CERAD-IR using fully adjusted weighted linear regression models.

	No Leisure-time PA	Insufficient Leisure-time PA	p	Sufficient Leisure-time PA	p
Cluster_braycurtis					
1	Reference	0.141(-0.624,0.907)	0.699	0.384(-0.267,1.035)	0.228
2	Reference	-0.699(-2.454,1.056)	0.331	0.066(-1.698,1.830)	0.922
3	Reference	0.585(-0.133,1.302)	0.103	0.33(-0.045,0.705)	0.081
4	Reference	0.493(-3.624,4.610)	0.658	-0.001(-4.019,4.016)	0.999
Cluster_unwunifrac					
1	Reference	-0.365(-1.483,0.753)	0.488	0.21(-0.865,1.285)	0.646
2	Reference	0.448(-0.135,1.031)	0.122	0.311(-0.160,0.782)	0.179
3	Reference	0.378(-0.035,0.953)	0.062	0.663(-2.088,3.414)	0.489
4	Reference	0.058(-0.767,0.884)	0.882	0.154(-0.479,0.788)	0.611
Cluster_wunifrac					
1	Reference	1.174(-Inf,Inf)	0.232	1.154(-Inf,Inf)	0.374
2	Reference	0.177(-0.560,0.914)	0.616	0.229(-0.488,0.947)	0.506
3	Reference	0.421(-0.349,1.191)	0.262	0.338(-0.270,0.946)	0.255
4	Reference	-0.103(-3.150,2.944)	0.921	-0.032(-1.294,1.230)	0.941

Table S8. Moderator effect of three β-diversity measures on leisure-time physical activity and CERAD-DR using fully adjusted weighted linear regression models.

	No Leisure-time PA	Insufficient Leisure-time PA	p	Sufficient Leisure-time PA	p
Cluster_braycurtis					
1	Reference	-0.6(-1.833,0.632)	0.316	0.57(-0.135,1.275)	0.105
2	Reference	-0.754(-1.800,0.291)	0.123	0.166(-2.217,2.549)	0.865
3	Reference	0.516(-0.969,2.000)	0.470	0.699(-0.206,1.604)	0.120
4	Reference	-0.11(-5.557,5.337)	0.939	-1.644(-6.687,3.400)	0.296
Cluster_unwunifrac					
1	Reference	-0.439(-4.076,3.199)	0.767	0.334(-2.712,3.380)	0.786
2	Reference	-0.091(-1.206,1.025)	0.865	0.254(-0.525,1.032)	0.498
3	Reference	-0.543(-3.247,0.892)	0.265	2.224(0.544,3.904)	0.029
4	Reference	-0.274(-1.699,1.151)	0.687	0.747(-0.163,1.658)	0.101
Cluster_wunifrac					
1	Reference	1.092(-2.413,4.598)	0.312	0.265(-4.227,4.757)	0.823
2	Reference	-0.688(-2.138,0.762)	0.328	0.174(-1.059,1.408)	0.768
3	Reference	0.442(-0.540,1.424)	0.353	1.069(0.431,1.707)	0.003
4	Reference	-0.7(-5.898,4.498)	0.697	-1.425(-5.002,2.152)	0.294

Table S9. Moderator effect of three β-diversity measures on leisure-time physical activity and AFT using fully adjusted weighted linear regression models.

	No Leisure-time PA	Insufficient Leisure-time PA	p	Sufficient Leisure-time PA	p
Cluster_braycurtis					
1	Reference	1.877(-1.270,5.024)	0.223	2.928(0.723,5.132)	0.013
2	Reference	-8.026(-14.683,-1.369)	0.027	0.744(-7.661, 9.149)	0.829
3	Reference	3.396(0.629,6.162)	0.019	0.833(-1.422,3.088)	0.443
4	Reference	9.315(-3.183,21.813)	0.085	14.601(6.965,22.236)	0.014
Cluster_unwunifrac					
1	Reference	2.354(-4.914,9.622)	0.490	-3.027(-10.099,4.044)	0.403
2	Reference	1.64(-0.051,3.330)	0.056	2.12(0.573,3.666)	0.011
3	Reference	1.456(0.237,4.263)	0.024	3.305(-10.546,17.156)	0.413
4	Reference	5.171(-0.788,11.129)	0.084	2.064(-3.600, 7.728)	0.449
Cluster_wunifrac					
1	Reference	9.722(-4.920,24.364)	0.104	6.639(-2.912,16.189)	0.096
2	Reference	2.66(-0.452,5.773)	0.088	1.711(0.276,3.146)	0.023
3	Reference	0.792(-1.510,3.094)	0.475	1.747(-2.391,5.885)	0.382
4	Reference	0.054(-3.951, 4.060)	0.968	7.798(-1.209,16.804)	0.070

Table S10. Moderator effect of three β-diversity measures on leisure-time physical activity and DSST using fully adjusted weighted linear regression models.

	No Leisure-time PA	Insufficient Leisure-time PA	p	Sufficient Leisure-time PA	p
Cluster_braycurtis					
1	Reference	3.118(-6.209,12.446)	0.487	6.834(0.139,13.528)	0.046
2	Reference	-2.697(-26.407,21.013)	0.768	14.143(-12.055,40.341)	0.208
3	Reference	6.121(-4.009,16.251)	0.217	7.527(0.761,14.293)	0.032
4	Reference	4.364(-60.756,69.484)	0.800	9.879(-47.689,67.448)	0.537
Cluster_unwunifrac					
1	Reference	-22.755(-55.340, 9.831)	0.269	-12.403(-39.739,14.933)	0.386
2	Reference	6.997(-0.327,14.322)	0.060	6.252(1.230,11.274)	0.018
3	Reference	5.833(0.233,12.315)	0.029	10.518(-19.132,40.168)	0.266
4	Reference	0.906(-4.563, 6.375)	0.729	10.893(2.530,19.256)	0.014
Cluster_wunifrac					
1	Reference	3.168(-22.770,29.107)	0.652	9.502(-14.058,33.062)	0.225
2	Reference	2.109(-7.559,11.777)	0.649	5.976(-0.753,12.706)	0.078
3	Reference	7.883(0.755,15.011)	0.032	9.073(1.863,16.284)	0.017
4	Reference	-17.257(-70.606,36.093)	0.379	5.672(-34.929,46.274)	0.687

Table S11. Association results of leisure time physical activity and white blood cell count using weighted linear regression models

	Crude model		Adjusted Model	
	β (95% CI)	P-value	β (95% CI)	P-value
Continuous LTPA (Per 100-MET increase)	-0.023 (-0.038, -0.007)	0.004	-0.025 (-0.040, -0.009)	0.002
Category LTPA				
No	Reference		Reference	
Insufficient (1-600 MET)	-0.416(-1.104, 0.272)	0.217	-0.328(-0.869, 0.214)	0.212
Sufficient (> 600 MET)	-0.883(-1.552,-0.214)	0.013	-0.923(-1.642,-0.204)	0.016

Notes: Crude model, no covariates were adjusted. Adjusted Model, age, sex, race/ethnicity, body mass index, marital status, education, poverty income ratio, smoke status, alcohol use status, and chronic diseases were adjusted.

Table S12. Association results of four alpha-diversity metrics, three beta-diversity measures and white blood cell count using weighted linear regression models

		Crude model		Adjusted Model	
		β (95% CI)	P-value	β (95% CI)	P-value
OTUs	Continuous	0.001(-0.008,0.009)	0.867	-0.001(-0.008, 0.007)	0.833
	Q1 (<80.20)	Reference		Reference	
	Q2 (80.20-101.08)	0.039(-0.893,0.971)	0.931	0.028(-0.817, 0.873)	0.944
	Q3 (101.08-126.71)	0.221(-0.372,0.814)	0.438	0.140(-0.487, 0.766)	0.634
	Q4 (>126.71)	0.133(-0.657,0.923)	0.724	0.021(-0.678, 0.720)	0.949
FaPhyloDiv	Continuous	0.036(-0.081,0.153)	0.526	0.021(-0.097, 0.139)	0.728
	Q1 (<10.58)	Reference		Reference	
	Q2 (10.58-12.32)	0.263(-0.495,1.021)	0.469	0.243(-0.467, 0.954)	0.467
	Q3 (12.32-14.34)	0.069(-0.580,0.717)	0.824	-0.079(-0.768, 0.610)	0.805
	Q4 (>14.34)	0.370(-0.487,1.227)	0.370	0.269(-0.566, 1.105)	0.492
InvSimpson	Continuous	-5.360(-9.337,-1.383)	0.011	-6.045(-10.196,-1.893)	0.008
	Q1 (<0.88)	Reference		Reference	
	Q2 (0.88-0.91)	-0.083(-0.963,0.797)	0.842	-0.148(-0.956, 0.660)	0.695
	Q3 (0.91-0.93)	0.015(-0.548,0.578)	0.849	-0.009(-0.718, 0.700)	0.978
	Q4 (>0.93)	-0.645(-1.343,0.054)	0.068	-0.704(-1.330,-0.078)	0.031
ShanWienDiv	Continuous	-0.324(-0.725,0.077)	0.106	-0.381(-0.754,-0.009)	0.045
	Q1 (<4.09)	Reference		Reference	
	Q2 (4.09-4.49)	-0.356(-1.001,0.289)	0.256	-0.421(-1.040, 0.198)	0.163
	Q3 (4.49-4.93)	-0.135(-0.575,0.305)	0.522	-0.178(-0.539, 0.183)	0.301
	Q4 (>4.93)	-0.515(-1.182,0.151)	0.119	-0.596(-1.155,-0.038)	0.039

Braycurtis distance	Cluster 1	Reference		Reference	
	Cluster 2	-0.363(-1.554,0.829)	0.524	-0.581(-1.824, 0.662)	0.325
	Cluster 3	0.135(-0.490,0.761)	0.650	0.111(-0.553, 0.775)	0.720
	Cluster 4	0.121(-0.736,0.979)	0.766	0.332(-0.595, 1.260)	0.447
Unweighed Unifrac	Cluster 1	Reference		Reference	
	Cluster 2	-1.821(-3.497,-0.145)	0.035	-1.851(-3.289,-0.413)	0.016
	Cluster 3	-2.055(-3.262,-0.849)	0.003	-2.041(-2.622,-1.460)	<0.001
	Cluster 4	-1.554(-3.561, 0.454)	0.119	-1.736(-3.438,-0.034)	0.046
Weighed Unifrac	Cluster 1	Reference		Reference	
	Cluster 2	0.189(-1.185,1.563)	0.772	0.154(-1.272, 1.580)	0.817
	Cluster 3	-0.005(-1.313,1.303)	0.993	-0.021(-1.295, 1.254)	0.972
	Cluster 4	-0.441(-2.162,1.281)	0.591	-0.567(-2.474, 1.339)	0.526

Notes: Crude model, no covariates were adjusted. Adjusted Model, age, sex, race/ethnicity, body mass index, marital status, education, poverty income ratio, smoke status, alcohol use status, and chronic diseases were adjusted.

Table S13. Association results of white blood cell count and cognitive function tests using weighted linear regression models

	Crude model		Adjusted Model	
	β (95% CI)	P-value	β (95% CI)	P-value
CERAD-IR	-0.025 (-0.088, 0.038)	0.431	-0.011 (-0.072, 0.050)	0.725
CERAD-DR	-0.047 (-0.137, 0.043)	0.305	-0.024 (-0.111, 0.062)	0.579
AFT	-0.003 (-0.240, 0.234)	0.981	0.007 (-0.229, 0.244)	0.950
DSST	-0.901 (-1.651, -0.151)	0.019	-0.763 (-1.494, -0.032)	0.041

Notes: Crude model, no covariates were adjusted. Adjusted Model, age, sex, race/ethnicity, body mass index, marital status, education, poverty income ratio, smoke status, alcohol use status, and chronic diseases were adjusted.
